# Cardiopulmonary Cement Embolism Following Cement-Augmented Pedicle Screw Fixation: A Narrative Review

**DOI:** 10.3390/medicina59020407

**Published:** 2023-02-19

**Authors:** Tadatsugu Morimoto, Takaomi Kobayashi, Hirohito Hirata, Masatsugu Tsukamoto, Tomohito Yoshihara, Yu Toda, Masaaki Mawatari

**Affiliations:** Department of Orthopaedic Surgery, Faculty of Medicine, Saga University, 5-1-1 Nabeshima, Saga 849-8501, Japan

**Keywords:** fenestrated pedicle screw, cement-augmented fenestrated pedicle screw, cement-augmented pedicle screw, cardiopulmonary embolism, pulmonary embolism, cement embolism

## Abstract

Fixation using cement-augmented pedicle screws (CAPS) is being increasingly performed. However, CAPS-associated cement leakage is a critical problem that can lead to cardiopulmonary cement embolism (CPCE). This narrative review aimed to explore the incidence of and risk factors and treatment strategies for CPCE and cement leakage-related complications after CAPS fixation. Data were extracted from each article, including characteristics of CPCE after CAPS fixation (incidence, location, diagnostic method and criteria, treatment, and outcome and prognosis). Overall, 28 case series and 14 case reports that met the inclusion criteria were included. Of the 1974 cases included in the review, CPCE was noted in 123, symptomatic CPCE in 35, and death in six, respectively. The frequencies of PCE and symptomatic PCE after CAPS fixation were 6% (range: 0–28.6%) and 1.3% (range: 0–26%), respectively. The range of frequencies of PCE and symptomatic PCE after CAPS fixation may have been wide because the definition of CPCE and data collection methods differed among the reports analyzed. Since PCE due to large cement emboli may be primarily related to the surgical technique, improved technique, such as minimizing the number of CAPSs by injecting low-volume high-viscosity cement at low velocity and pressure, and careful observation of cement leakage during CAPS insertion may reduce PCE associated with cement leakage. Spinal surgeons should pay more attention to the occurrence of CPCE during and after CAPS insertion, which can cause serious complications in some patients.

## 1. Introduction

Extended life expectancy and improved quality of life have increased the number of older patients with osteoporosis undergoing spinal surgery [1]. In osteoporotic spines, sufficient fixation strength has not been achieved because of the loss of healthy bone structure due to aging. Osteoporosis-related implant failures can be difficult to treat, thereby burdening patients and surgeons and significantly impacting healthcare economics. Therefore, methods to improve screw fixation need to be established. Over the past few decades, several techniques to increase the anchoring strength of pedicle screws and reduce the risk of screw loosening have been developed, with cement-augmented pedicle screws (CAPS) being the most promising and beginning to be widely used. The use of CAPS in patients with osteoporosis strengthens screw fixation, decreases the incidence of screw loosening, and improves the fusion rate [2,3], thereby possibly reducing the extent of spinal fusion and directly contributing to minimizing surgical invasiveness; therefore, CAPS is consistent with the concept of minimally invasive spinal treatment.

However, while CAPS is being increasingly used, post-CAPS cement leakage is a considerable problem. Cement leakage into the spinal canal can cause spinal cord compression symptoms, including neurologic deficits and pain, while leakage into the epidural vein, vena cava, right atrium, pulmonary artery, or multiple lung arterioles can cause cardiac or pulmonary cement embolism (PCE) [2,3,4,5,6,7,8,9,10,11,12,13,14,15,16,17]. Since both embolisms have the same pathology, this study included cardiopulmonary cement embolisms after CAPS as CPCE. In addition, bone cement can cause an anaphylactic reaction, leading to shock and death. Heat associated with cement polymerization can also cause neurological damage [18]. However, only a few publications have reported the incidence of CPCE or CAPS-related complications, most in the form of case reports [2,4,5,6,7,8,9,10,11,12,13,14,15,16,17]. CPCE rates reported in past studies showed a wide prevalence range because it does not have a standard definition, and different studies used different methods to evaluate cement embolism [2].

Furthermore, the level of evidence from existing studies regarding CPCE after CAPS has been relatively low [2]. Hence, management protocols for post-CAPS CPCE remain unknown. This narrative review aimed to explore the incidence of and risk factors for CPCE and cement leakage-related complications after CAPS and to provide evidence for treatment strategies for CPCE after CAPS.

## 2. Methods

**Figure 1:**
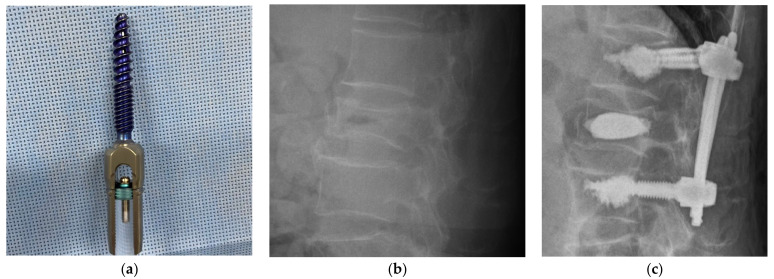
Fenestrated screws (**a**) Expedium Verse^Ⓡ^ spinal system and Vertecem V+^Ⓡ^ cement (DePuy Synthes Products, Inc., Raynham, MA, USA), (**b**) Case illustration: preoperative lumbar X-ray, 82-year-old man with L1 vertebral fracture pseudarthrosis in the osteoporotic spine (T= −2SD). (**c**) Case illustration: postoperative lumbar X-ray. L1vertebroplasty and T12-L2 posterior fusion with cement-augmented fenestrated screws was performed.

Two major types of CAPS were found depending on the screw type:

(1) Conventional solid screws: cement-brushed solid screw inserted [19] or solid screw inserted after vertebroplasty [4,5,6,7,8,9,10,20,21,22,23].
(2) Fenestrated screws (Figure 1): these can be cemented after screw placement [11,12,13,14,15,24,25,26,27,28,29,30,31,32,33,34,35,36,37,38,39,40,41,42,43,44,45,46].

A narrative review based on PRISMA guidelines was performed by two independent reviewers using the Cochrane Library and PubMed databases between January 2000 and October 2022 [47,48]. Both MeSH terms and free-text keywords were utilized for searching for relevant articles without setting a minimum or maximum postoperative follow-up window. The search terms “fenestrated pedicle screw,” “cement-augmented fenestrated pedicle screw,” “cement-augmented pedicle screw,” “pulmonary embolus,” and “cement embolus” were applied to identify 56 available records for further evaluation. Case reports were also included to cover currently available information on the frequency, symptoms, and treatment of PE or PCE after CAPS fixation. In order to avoid overlooking additional studies, we searched the bibliography of selected articles. Data from the included articles were independently extracted by two reviewers. Data extracted from each article included baseline data (first author, year of publication, study type, sample size, country, level of manipulation, sex, age, solid or fenestrated screw, and amount of cement) and characteristics of PE after CAPS fixation (incidence, location, diagnostic method and criteria, treatment, and outcome and prognosis).

Studies investigating the presence of PE as an outcome or case reports of PE after spinal fusion with CAPS were included. The indications for surgery were open-ended to include all types of degenerative spinal conditions and fractures.

The following studies were excluded: Studies regarding patients undergoing spinal fusion with conventional screws without cement augmentation; review articles, non-English publications, and studies for which full texts were unavailable; in cases of data duplication, studies with the largest sample size were included and the rest excluded.

## 3. Results

This study included 28 case series and 14 case reports that met the inclusion criteria. Of the 1974 included cases (1960 from the case series and 14 from case reports), CPCE was noted in 123 (114 and 9), symptomatic CPCE in 35 (26 and 9), and death in six (5 and 1), respectively.

### 3.1. Characterisitics of CPCE after CAPS Fixation from the Case Studies

From the 28 case series studies (three prospective and 23 retrospective studies), PCE occurred in 6% (114/1960) of all patients with CAPS, 5.3% (22/419) with conventional solid screws, and 6% (81/1339) with fenestrated screws (Table 1).

Symptomatic PCE occurred in 1.3% (26/1960) of all patients after CAPS fixation, 3.1% (13/419) with conventional solid screws, and 0.8% (11/1339) with fenestrated screws.

The distribution of PCE incidence in all cases was 0–28.6% (<5%: 22 studies, 5–10%: five studies, 11–20%: one study, >20%: two studies) and 0–26% (<5%: 25 studies, 5–10%: none, 11–20%: none, >20%: one study, not available: one study), respectively. No cardiac embolism was observed.

Regarding the years reported, conventional solid screws were common from 2009 to 2017, while fenestrated screws were increasingly reported after 2018.

PCE diagnosis was evaluated by chest radiography and computed tomography (CT) in all cases in one article [34], chest CT in all cases in two [19,27], chest radiography in all cases in three [24,25,31], chest radiography in all cases and chest CT in selected patients in two [23,35], chest CT in selected patients in six [20,22,38,39,40,46], chest radiography or CT in selected cases in one [33], chest radiography and intraoperative transesophageal echo in one [41], and no clear description in 12 articles [21,26,28,29,30,32,36,37,42,43,44,45]. Seven patients required cardiopulmonary resuscitation (CPR) due to shock [23,34], and five patients died (four due to PE and one due to suspected cement-induced anaphylactic shock) [22,32,35,42].

### 3.2. Characteristics of CPCE after CAPS Placement from the Case Reports

Of the 14 case reports, conventional solid type screws were reported in seven studies [4,5,6,7,8,9,10], fenestrated screws in five [11,12,13,14,15], and the details were unknown in two [16,17] (Table 2). In terms of reporting years, six of the seven case reports on conventional solid screws were reported between 2010 and 2013, while four of the five case reports regarding fenestrated screws were reported after 2020. Regarding the number of CAPS inserted, four screws were inserted in seven studies [5,6,7,11,12,13,15], six in two [9,14], eight and 10 in one case report each [10,17], and the number was unknown in three [4,8,16]. Time of embolism detection was during surgery in three cases [4,15,17]; immediately after surgery in four [7,8,13,16]; 1 day after in two [10,11]; 2, 3, and 6 days after surgery in one case each [5,12,14]; and unknown in two [6,9]. Clinical presentation comprised cardiopulmonary symptoms (tachycardia, hypoxia, dyspnea, chest pain) in nine patients [5,7,8,10,11,12,13,14,16], two of whom required CPR [8,10]; asymptomatic presentation during surgery in three cases [4,15,17], and unknown details in two cases [6,9]. Cement leakage from the vertebral body into the inferior vena cava (IVC) was bilateral in two cases [13,17], right-sided in four [4,9,14,15], and unknown in eight [5,6,7,8,10,11,12,16]. PE was reported in 12 cases [4,5,6,7,8,9,11,12,13,14,16,17] and cardiac (right atrial) embolism in two [10,15]. Management included anticoagulation in five cases [4,7,9,11,13], CPR in two [8,10], cement removal with a catheter using an endovascular approach in two [5,15], surgical cement removal in two [12,17], none and unknown in one [6], with death reported in one case [8] (Table 2).

## 4. Discussion

### 4.1. Rate and Diagnosis of CPCE after CAPS Fixation in the Case Series

In the CPCE case series, all cases were of PCE, with none reporting cardiac cement embolisms. The frequency of PCE after CAPS insertion in 28 studies (1960 cases) was 6% (114 cases) (range: 0–28.6%), of which 1.3% (26 cases) (range: 0–26%) was symptomatic.

The incidences of PCE and symptomatic PCE ranged from 0–28.6% and 0–26%, respectively, with both being widely distributed. The incidence of PCE after percutaneous vertebroplasty was reported as 2.3% (144/6251 cases) (range: 0–25%) in a systemic review, but the range was as wide as that in this study, presumably due to different study methods, etiology, and diagnostic criteria [49]. However, an incidence rate < 5% was noted in 22/28 studies regarding PCE and 25/28 studies regarding symptomatic PCE, a relatively low frequency, similar to that reported by Yagi et al. [2]. This could be attributed to the retrospective design in most (25/28) of the studies and their unclear definitions for PCE and underestimated incidence rates. Additionally, only three studies performed routine CT examinations for PCE diagnosis in all patients. Studies on the rate of PCE after percutaneous vertebroplasty have reported a radiographic diagnostic rate of 6.8% and a CT rate of 23% [50,51]. CT is highly sensitive in detecting PCE, especially peripheral PCEs of less than 4 mm in diameter [51]. Thus, we speculated that the wide range of PCE rates in our study and relatively low rates in most previous ones could be attributed to unclear PCE definitions and varying methods for PCE assessment among the studies reviewed (with only three studies utilizing routine CT scans). Therefore, the PCE rate was likely underestimated in most previous reports. On the other hand, the low frequency of symptomatic PCE and concerns regarding radiation exposure justify the need for postoperative chest radiography, even in asymptomatic patients. However, whether a CT scan with greater sensitivity to diagnose PCE is justified as a screening procedure remains unclear, similar to the discussion by Krueger et al. regarding the pros and cons of performing chest CT for PCE after percutaneous vertebroplasty and kyphoplasty [52].

According to the screw type, PCE incidence was similar for conventional solid (5.3%) and fenestrated (6%) screws. With respect to reporting years, conventional solid screws were reported more frequently before 2017, while fenestrated screws were increasingly reported after 2018. Although using conventional solid screws may be less expensive than using fenestrated screws, they may complicate the procedure. Once we begin inserting the solid screw into viscous cement, there is limited control over dealing with any leaks. If a leak occurs, the surgeon must decide whether to continue implantation or stop and pull the screw out completely, despite the continued leak [27]. Thus, since its introduction, the fenestrated pedicle screw has been widely used and continues to increase in popularity.

### 4.2. Symptoms of CPCE after CAPS Insertion

Symptoms are subclinical in most cases of PCE. The clinical picture of symptomatic PCE resembles that of a thrombotic PE: it is characterized by tachycardia, dyspnea, hypotension, loss of consciousness, and can lead to cardiopulmonary arrest and death.

Several studies have demonstrated postoperative lung injury with inflammatory features resulting from pulmonary cement deposition in patients undergoing cemented knee arthroplasty [53,54,55]. Most patients with osteoporosis eligible for CAPS were older adults, and older age was a risk factor for respiratory complications [41]. Additionally, since respiratory complications in older patients are a major cause of longer hospital stays and perioperative morbidity and mortality [56,57], symptomatic PCE with possible lung injury should be monitored for. On the other hand, cardiac cement embolism after CAPS fixation was reported in two cases in this study: one with chest pain [10] and the other diagnosed during CAPS insertion (without symptom presentation under anesthesia) [15]. Similar to our study, a significant number of case reports regarding PCE after percutaneous vertebroplasty have been reviewed; however, only a few isolated case reports regarding cardiac cement embolism exist in the literature, which have not been previously reviewed [58]. In cardiac cement embolism, cardiac perforation due to sharp cement fragments may necessitate pericardiocentesis or excision, and concomitant valvular disease with cement embolization may require additional valve replacement surgery, depending on the degree of regurgitation [58].

### 4.3. Pathology of CPCE after CAPS Placement

Three distinct pathological outcomes of symptomatic PCE after percutaneous vertebroplasty have been observed: (1) mechanical obstruction of the heart and lungs by cement fragments, (2) fat embolism syndrome caused by displacement of bone marrow particles into the vascular system by the injected cement, and (3) anaphylactic reaction to the cement [49].

(1) Mechanical obstruction

The most common outcome is mechanical obstruction of the heart or lungs by cement fragments. The hydroxyapatite and allograft bone, used as additional reinforcements to strengthen the pedicle screw fixation, can also cause PE due to fat and bone marrow particles as well as from debris of material augmentation (hydroxyapatite and allograft bone) during screw insertion [59,60].

(2) Fat embolism

Fatal fat embolism syndrome associated with isolated osteoporotic vertebral fractures [61], vertebroplasty [62], and simple pedicle screw fixation without augmentation hs been reported [63]. Both vertebral fractures and spinal surgeries can involve the bone marrow, increasing the intraosseous pressure, which dislodges fat and bone marrow contents into venous circulation, thereby causing systematic inflammation [59].

“Echogenic material,” reflecting bone marrow and fat, has been observed passing through the right atrium using echocardiography during the following steps of spinal surgery: (1) probing of the vertebral body [63], (2) placement of the pedicle screw [64,65], (3) insertion of hemostatic agents into the pedicle screw pilot hole [65,66], and (4) cement insertion during vertebroplasty [67] and fenestrated pedicle screw augmentation [41].

Although less frequent than mechanical obstruction, fatal cases of fat embolism syndrome without cement embolism after CAPS fixation [62] and death due to an anaphylactic reaction to cement [22] have been reported; therefore, spinal surgeons should pay attention to these conditions when managing patients during and after CAPS insertion.

### 4.4. Risk Factors for CPCE after CAPS Fixation

With regards screw type, CPCE may be mainly caused by the vertebroplasty procedure before screw placement for conventional solid type screws, and by the cement insertion procedure after screw placement for fenestrated screws. Despite only a few available studies regarding risk factors for embolism after CAPS fixation, the anatomical and technical aspects have been examined.

#### 4.4.1. Anatomical Aspect

Some reports found that patients who received instrumentation in the thoracic or thoracolumbar spine were at significantly higher risk for PCE than those who received it in the lumbar spine [22,34]. A review of percutaneous vertebroplasty also found a higher incidence of PCE at the thoracic level than at the lumbar level. This could be possibly owing to the fact that in comparison with the lumbar bodies, thoracic vertebral bodies are smaller in size and closer to the cardiopulmonary vessels [49]. Although the number of studies (number of cases) reporting this issue are limited, this may be an anatomical note to keep in mind when performing CAPS. The relationship between the IVC and lumbar vein and characteristics of the lumbar vein need to be discerned, because intraoperative cement leakage into the IVC significantly contributes to PCE [68]. The lumbar veins enter the IVC at the L1-L5 vertebral positions, and cement flow into the IVC may result from the numerous connections to the vertebral and branch veins, lack of valves in the internal and external venous plexus, marked venous enlargement in older individuals, and lower pressure in the vertebral venous system than in the pelvic veins [34,68,69]. Iwanaga et al. demonstrated that latex or air injections into the lumbar vertebral bodies drain specifically into the IVC and not internally into the vertebral venous plexus within the vertebral canal [70]. This indicates that cement injection can similarly enter the IVC; therefore, the close relationship between the IVC and lumbar vein could be an anatomical risk factor for the occurrence of venous cement leakage. In addition, Guo et al. [46] noted that a right-sided approach was a risk factor, because leakage into the IVC after CAPS insertion was more common on the right side, supported by the anatomical location of the IVC anterior to the right of the lumbar vertebral body. In the current study, cement leakage from the vertebral body into the IVC was identified in two bilateral and five right-sided cases from 12 case reports regarding CPCE after CAPS fixation (with five unknown) (Table 2). Due to the anatomic configuration of the vertebral body and IVC, attention should be paid when cementing from the right anterior side during CAPS insertion.

#### 4.4.2. Technical Aspect

Similar to percutaneous vertebroplasty, high cementing pressure, low cement viscosity, high cementing volume, and increased number of CAPS during CAPS insertion have been noted to be closely correlated with cement leakage and are reportedly risk factors for PCE [31,34].

Cement embolisms are more likely to occur when low-viscosity cement is injected at high pressure [31,34]. On the other hand, Frankel et al. reported no relationship between the number of CAPS used and PCE [24]. In this study, we reviewed case reports of symptomatic PCE; seven out of 14 cases utilized four CAPS with only the upper and lower ends fixed, and there was a risk of PCE even with minimal use of CAPS. Although selective cement reinforcement of cephalic and caudal pedicle screws appears to be a valuable strategy for reducing complications, the experience and knowledge of the surgeon, including not using low-viscosity cement and avoiding high-pressure injection, may influence the PCE risk more than the number of CAPS used.

### 4.5. Management of CPCE after CAPS Fixation

For prevention of CPCE after CAPS fixation, procedural precautions should include adequate fluoroscopy using a good-quality biplane fluoroscopy device to confirm the extent and direction of cement injection, careful use of the correct tap position, discontinuation of cement injection if any extra vertebral leakage is suspected, and performance by an experienced surgeon [41]. Considering the risk factors for cement leakage, minimizing the number of CAPS used by injecting high-viscosity, low-volume cement at low speed, and pressure is recommended [41]. From an anatomical standpoint, special attention should be paid to the CAPS placed within the right anterior section of the vertebral body, proximal to the IVC. Since PCE due to large cement emboli may be primarily related to aspects of the surgical approach, an improved surgical technique can potentially reduce cement leakage and the associated PCE. In the case reports reviewed in this study, most of the symptomatic CPCEs were detected during or immediately after CAPS insertion. In addition, fat embolism was also observed by echocardiography at the time of cement injection [41]. Therefore, aside from spinal surgeons, anesthesiologists must carefully note any sudden decrease in arterial blood pressure, oxygen saturation, or carbon dioxide concentration during or after cementation, which may be indicators of CPCE.

Although no clear guidelines for the management of PCE exist in the literature, it is generally agreed that treatment should be based on the presenting symptoms and location of the embolism [9,52,69].

For asymptomatic PCE, clinical follow-up without anticoagulant prescription has been recommended by some authors [51,71]. For symptomatic cases, initial anticoagulation with heparin and follow-up with coumarin therapy for 6 months has been suggested [5,7,11,13,16,17].

Emergency cardiovascular surgery, including interventional radiology or open/minimally invasive cardiac surgery, may be required in cases of main artery invasion or PCE trapped within the atrium [5,12,14,15,17]. Large cement emboli trapped within the pulmonary artery or atrium can sometimes be retrieved with endovascular procedures performed under fluoroscopy [5,15]. While percutaneous removal is an attractive procedure, open cardiovascular surgery may still be necessary for complete removal in cases of atrial perforation by cement fragments or large PCE [12,14,17]. Therefore, strict indications for CAPS implementation are necessary to minimize risk, because CAPS fixation is not an entirely safe procedure, particularly for patients with osteoporosis and concomitant cardiac or respiratory disease. CAPS should also be used with caution, especially in patients with contraindications to anticoagulation or endovascular therapy or open cardiovascular surgery.

This study had some limitations. First, since we only included publications written in English, a language bias may exist. Second, the diagnosis of PCE (especially asymptomatic PCE) may not have been standardized among the included reports. Finally, although vertebral fracture type, such as AO spine classification of thoracolumbar injuries [72]) may also be a risk factor for CAPS, this was not studied. Larger prospective studies need to be conducted to analyze whether thoracic level or vertebral fracture type could be independent risk factors for CAPS. Despite these limitations due to the nature of the literature review, our findings can potentially contribute to clinical practice.

## 5. Conclusions

The frequencies of PCE and symptomatic PCE after CAPS fixation are 6% (range: 0–28.6%) and 1.3% (range: 0–26%), respectively, with both being widely distributed. The definition of CPCE and method of data collection varied among the analyzed reports, which may have resulted in the wide range of frequencies. Since PCE due to large cement emboli may be primarily related to the surgical technique, improved technique, such as minimizing the number of CAPSs by injecting low-volume, high-viscosity cement at low velocity and pressure, and careful observation of cement leakage during CAPS insertion may reduce the PCE associated with cement leakage. Spinal surgeons should pay more attention to the occurrence of CPCE during and after CAPS insertion, which can cause serious complications in a minority of patients. Additional large-scale multicenter studies may be required to obtain more generalizable results of higher quality.

## Figures and Tables

**Table 1:** Summary of study characteristics.

Authors, Year	Nationality	Study Design	Sample	Men: Women	Mean Age (Years)	Number of CAPS Used	Evaluation Method for PCE	Incidence of PCE n (%)	No of Symptomatic PCE n (%)	Symptoms, Progression, and Treatment of PCE	Fatal Cases (Cause)
Conventional solid screw fixation											
Aydogan et al., 2009 [20]	Turkey	Retrospective	49	12:24	66 (59 to 78)	NA	Chest CT in selected patients (oxygen saturation <95% at room air)	1(2%)	0	NA	0
Sawakami et al., 2012 [19]	Japan	Retrospective	17	5:12	73.8	NA	Chest CT in all patients	0	0	NA	0
Seo et al., 2012 [21]	Korea	Retrospective	157	49:108	66.5(49 to 74)	947	NA	0	0	NA	0
Janssen et al., 2017 [22]	Germany	Retrospective	165	62:103	71 (46 to 93)	1330	Chest CT in selected patients	13(7.9%)	5(3%)	Four patients experienced life-threatening hemodynamic reactions, cement embolism (*n* = 2) or anaphylactic shock (*n* = 2), and three required intraoperative CPR	2 (fulminant PCE)
Erdem et al., 2017 [23]	Turkey	Retrospective	31	5:26	NA	149	Chest radiography in all patients, Chest CT in selected patients	8(26%)	8(26%)	Eight patients had chest discomfort	0
Fenestrated screw fixation											
Frankel et al., 2007 [24]	USA	Retrospective	23	6:17	64.7	NA	Chest radiography in all patients	1(4.3%)	0	NA	0
Moon et al., 2009 [25]	Korea	Retrospective	37	2:37	68.7	168	Chest radiography in all patients	0	0	NA	0
Lubansu et al., 2012 [26]	Belgium	Prospective	15	3:12	71.2	78	NA	0	0	NA	0
El Saman et al., 2013 [27]	Germany	Retrospective	42	16:26	74 (57 to 89)	311	Chest CT in all patients	12(28.6%)	NA	No life-threatening symptoms	0
Pesenti et al., 2014 [28]	France	Retrospective	12	5:07	73	96	NA	1(2.7%)	0	NA	0
Klingler et al., 2015 [29]	Germany	Retrospective	35	10:25	72.8	85	NA	0	0	NA	0
Dai et al., 2015 [30]	China	Retrospective	43	13:30	67.7	NR	NA	0	0	NA	0
Mueller et al., 2016 [31]	Germany	Retrospective	98	38:60	70.6	474	Chest radiography in all patients	4(4%)	0	NA	0
Girardo et al., 2018 [32]	Italy	Retrospective	32	6:26	76.9	224	NA	1(3.1%)	1(3.1%)	NA	1(embolism)
Rong et al., 2018 [33]	China	Retrospective	28	9:19	60.5	161	Chest radiography or CT in selected patients	0	0	NA	0
Ulusoy et al., 2018 [34]	Turkey	Retrospective	281	77:204	70.5 (51 to 89)	2978	Chest radiography and CT in all patients	46(16.3%)	4(1.4%)	All four patients with symptomatic PCE required CPR	0
Ishak et al., 2019 [35]	USA	Retrospective	86	23 64	73.4	458	Chest radiography in all and chest CT in selected patients	4(5%)	2(2.3%)	Anticoagulation therapy	1(cement-induced anaphylactic shock)
Wang et al., 2019 [36]	China	Retrospective	128	29:99	60.7	418	NA	0	0	NA	0
Barzilai et al., 2019 [37]	USA	Retrospective	53	30 23	63.5	216	NA	3(6%)	0	NA	0
Gazzeri et al., 2020 [38]	Spain	Prospective	20	9:11	71.3 (60 to 79)	NA	Chest CT in selected patients	0	0	NA	0
Liu et al. 2020 [39]	China	Retrospective	23	9:14	63.3	85	Chest CT in selected patients	0	0	NA	0
Tang et al., 2020 [40]	China	Retrospective	46	7:39	70.6	336	Chest CT in selected patients	2(4.3%)	1(0.2%)	dyspnea and hypoxia, anticoagulation	0
Rodriguez-Arguisjuela et al., 2021 [41]	Spain	Prospective	25	11:14	76.2	NA	Chest radiography, TTE, and arterial blood gas in all patients	0	0	NA	0
Coniglio et al., 2021 [42]	Italy	Retrospective	163	58:105	71.3(65 to 82)	1109	NA	1(0.6%)	1(0.6%)	NA	1(embolism)
Massaad et al.,2021 [43]	USA	Retrospective	69	38 31	64.7	502	NA	1(1.4%)	0	NA	0
Ehresman et al., 2021 [44]	USA	Retrospective	38	18:20	67.9	252	NA	2(5.2%)	1(2.6%)	NA	0
Wagner A et al., 2021 [45]	Germany	Retrospective	42	16:26	74 (57 to 89)	293	NA	3(5.9%)	1(2%)	NA	0
Conventional solid screw and fenestrated screw											
Guo et al., 2019 [46]	China	Retrospective	202	24:178	6639	950	Chest CT in selected pts	11(4.7%)	2(1%)	One patient had dyspnea and one, tightness of the chest	0

Abbreviations: CAPS: cement-augmented pedicle screw, NA: not available, CPR: cardiopulmonary resuscitation, TTE: transesophageal echocardiography, CT: computed tomography, PCE: pulmonary cement embolism.

**Table 2:** Summary of reported cardiopulmonary embolism caused by cement-augmented pedicle screw.

Authors, Year	Age (Years)/sex	Augmented Vertebral Levels	Number of CAPS Used	Time of Embolism Detection	Clinical Presentation	Left-Right Difference in Cement Leakage from the Vertebrae to the Inferior Vena Cava	Localization of Cardiopulmonary Cement Embolism	Management	Outcome
Conventional solid type									
Akinola et al., 2010 [4]	76/M	L3-5	NA	during surgery	No symptom	Right	Bilateral pulmonary arteries in CT	Anticoagulation therapy	Good Recovery
Rasch et al., 2010 [5]	55/M	L3-4	4	2 days after surgery	Tachycardia, dyspnea, hypoxia	NA	Right pulmonary artery and upper left lung lobe in CT	Cement removal with catheter using endovascular approach	Good Recovery
Röllinghoff et al., 2010 [6]	69/M	T8-L1	4	NA	NA	NA	Multiple arterioles in both lungs in CT	NA	Good Recovery
Tonolini et al., 2012 [7]	75/F	L1-3	4	Immediately after surgery	Tachycardia, dyspnea, hypoxia	NA	Right pulmonary artery and upper right lung lobe in CT	Anticoagulation therapy	Good Recovery
Zheng et al., 2013 [8]	47/F	T1-4, T9	NA	Immediately after surgery	Dyspnea, hypotension, unconsciousness	NA	Multiple pulmonary arterioles during autopsy	CPR	Death
Ignacio et al., 2013 [9]	34/M	T12-L2	6	NA	NA	Right	Central pulmonary artery in CT	Anticoagulation therapy	Good Recovery
Rahimizadeh et al., 2020 [10]	Middle age/F	L2-S	8	1 day after surgery	Cardiopulmonary arrest	NA	Left pulmonary artery and middle left lung lobe in CT	CPR, anticoagulation therapy	Good Recovery
Fenestrated type									
Özalay et al., 2013 [11]	75/F	L3-5	4	1 day after surgery	slight fever, chest pain, breathing difficulty	NA	Right pulmonary artery and middle right lung lobe in CT	Anticoagulation therapy	Good Recovery
Hong et al., 2020 [12]	67/F	L4-5	4	6 days after surgery	chest pain	NA	Multiple arterioles in both lungs in CT and penetrating the right atrium	Surgical cement removal	Good Recovery
Gomez et al., 2021 [13]	64/F	T9-L1	4	Immediately after surgery	Hypoxia	Bilateral	Both pulmonary arteries in CT	Anticoagulation therapy	Good Recovery
Liang et al., 2021 [14]	67/M	L4-S1	6	3 days after surgery	Hypoxia	Right	Right pulmonary artery and lower left lung lobe in CT	Surgical cement removal	Good Recovery
Takahashi et al., 2021 [15]	75/F	T11-L2	4	during surgery	No symptom	Right	Migrating into the right atrium in transesophageal echocardiography	Cement removal with catheter using endovascular approach	Good Recovery
Unknown type									
Hemmer et al., 2015 [16]	64/M	L3-5	NA	Immediately after surgery	The patient felt a little winded	NA	Left pulmonary artery and upper left lung lobe in CT	None	Good Recovery
Andrä et al., 2017 [17]	62/F	T11-L3	10	during surgery	No symptom	Bilateral	Penetrating the right atrium in CT	Surgical cement removal	Good Recovery

Abbreviations: NA: not available, CPR: cardiopulmonary resuscitation, CAPS: cement-augmented pedicle screw, CT: computed tomography.

## Data Availability

Not applicable.
